# Reinvestigation of Carbohydrate Specificity of EBCA-1 Monoclonal Antibody Used for the Detection of *Candida* Mannan

**DOI:** 10.3390/jof7070504

**Published:** 2021-06-24

**Authors:** Vadim B. Krylov, Arsenii S. Solovev, Ilya A. Puchkin, Dmitry V. Yashunsky, Anna V. Antonets, Olga Y. Kutsevalova, Nikolay E. Nifantiev

**Affiliations:** 1 Laboratory of Glycoconjugate Chemistry, N.D. Zelinsky Institute of Organic Chemistry, Russian Academy of Sciencesa, Leninsky Prospect 47, 119991 Moscow, Russia; v_krylov@ioc.ac.ru (V.B.K.); a_solovey97@mail.ru (A.S.S.); ilya.puchkin@gmail.com (I.A.P.); yashunsky1959@yandex.ru (D.V.Y.); antnts@mail.ru (A.V.A.); 2 Medical Genetic Center, Rostov-on-Don State Medical University, Nakhichevansky, 29, 344022 Rostov-on-Don, Russia; 3 National Medical Research Center of Oncology, Laboratory of Clinical Microbiology, 14 Liniya Str., 63, 344037 Rostov-on-Don, Russia; olga_kutsevalova@mail.ru

**Keywords:** *candida*, diagnosis, mannan, monoclonal antibodies, glycoarray

## Abstract

Monoclonal antibody EBCA-1 is used in the sandwich immune assay for the detection of circulating *Candida* mannan in blood sera samples for the diagnosis of invasive candidiasis. To reinvestigate carbohydrate specificity of EBCA-1, a panel of biotinylated oligosaccharides structurally related to distinct fragments of *Candida* mannan were loaded onto a streptavidin-coated plate to form a glycoarray. Its use demonstrated that EBCA-1 recognizes the trisaccharide β-Man-(1→2)-α-Man-(1→2)-α-Man and not homo-α-(1→2)-linked pentamannoside, as was reported previously.

## 1. Introduction

Opportunistic yeasts of the genus *Candida* are the most common agents causing nosocomial fungal infections, and the fourth most common cause of nosocomial bloodstream infections (BSI) overall. Invasive candidiasis (IC) affects about 750,000 people worldwide with a case fatality rate of ~30–55% [1]. Growing *Candida* resistance is an urgent problem [2]. The IC diagnosis is still difficult due to the lack of specificity of the clinical symptoms and poor sensitivity of the cultural methods on blood samples up to only ~50% [3,4]. That is why the diagnosis of IC must combine direct mycological methods with other techniques. Antigen and antibody tests, β-D-glucan detection assays, *Candida* DNA detection by polymerase chain reaction (PCR), and other non-cultural tests have been entering clinical practice as additives to cultural methods for the last few decades [5]. β-D-Glucan is a common cell wall component of lots of fungi including *Candida* species. The FDA has approved a serum β-D-glucan assay (Fungitell^®^ assay) for the diagnosis of invasive fungal infections. It helps to predict possible invasive fungal infection which, despite positive results, are not specific for *Candida* infection. The overall sensitivity and specificity of the β-D-glucan test for diagnosing IC accounted for 75–80% and 80%, respectively [6]. *Candida* PCR assays of various blood fractions help to reduce time to diagnose IC, especially compared to cultural methods. The sensitivity and specificity of PCR for suspected IC were 95% and 92%, respectively, as was shown in a recent meta-analysis [7].

Innovative nonculture molecular diagnostic assays have alternatively been developed and evaluated for the rapid identification of *Candida* species directly from clinical samples. These include the T2Candida^®^ assay (T2Biosystems, Lexington, MA, USA) and a broad-range PCR-coupled electrospray ionization mass spectrometry platform (PCR/ESI-MS), peptide nucleic acid-fluorescent in situ hybridization or PNA-FISH, and matrix-assisted laser desorption/ionization time-of-flight mass spectrometry or MALDI-TOF MS [8,9,10]. The T2Candida^®^ assay is a molecular method that combines PCR with T2 magnetic resonance (T2MR). It is approved by the FDA for rapid diagnosis of IC caused by clinically relevant *Candida* species at minimal concentrations of 1–3 colony-forming units per milliliter (CFUs/mL) in whole-blood samples [8]. The advantages, utility, and limitations of the other cutting-edge techniques, as well as their combined use to assist in the diagnosis of this life-threatening and costly fungal infection including BSI and IC, are under discussion [10]. Nevertheless, serological methods remain significant adjunct to cultural methods.

The circulating mannan antigen in the bloodstream is considered as a diagnostic marker of invasive *Candida* infection. The commercial kit for mannan detection is the Platelia Candida Antigen Plus (Bio-Rad, France) which is based on a “sandwich”-type enzyme-linked immunosorbent assay (ELISA) and uses the monoclonal antibody EBCA-1 to the mannan from the *Candida* spp. cell wall.

This kit is characterized by moderate sensitivity (50–70%) which can be increased up to 70–100% by its use in combination with the anti-mannan antibody detection kit, the Platelia Candida Ab-Plus (Bio-Rad, France). In a meta-analysis [11] of 14 studies, the sensitivities and specificities of the mannan and anti-mannan test separately for IC were 58% and 93%, and 59% and 86%, respectively. Sensitivity and specificity for combined mannan and anti-mannan assays were 83% and 86%, respectively, with the best performance in patients with *C. albicans*, *C. glabrata*, or *C. tropicalis* infections. Significant heterogeneity of studies was noted in the meta-analysis [11].

The mannan and anti-mannan tests are employed at many European centers but are not widely used in North America, as they are not cleared by the U.S. Food and Drug Administration [12]. In addition, according to the Clinical Practice Guideline for the Management of Candidiasis of the Infectious Diseases Society of America (updated in 2016), the role of mannan and anti-mannan in detecting antibodies remains unclear [5]. In the study including patients from an intensive care unit with severe abdominal pathology, the combined determination of mannan antigen and mannan antibodies to anti-mannan was ineffective (with sensitivity at 55% and specificity at 60%). Antibodies are often present in immunocompromised patients with preexisting candidemia or severe colonization [13]. Thus, the positive predictive value of detecting antibodies with a single test in the absence of subsequent detection of their increasing concentration remains low. This observation and the unexplained variability of tests across studies is an important caution for physicians, as inaccurate laboratory results can lead to inappropriate prescription of antifungal drugs to patients who are unlikely to have IC [14,15].

Such a controversial experience in the application of existing diagnostic tests that utilize the mannan marker leads to a range of questions. There were concerns over the use of the mannan as a diagnostic tool due to its natural colonization with *Candida*. Nevertheless, under these conditions, the mannan level remains within the cut-off, while it is greatly elevated in patients with IC [16]. Consequently, mannan remains a promising biomarker in the comprehensive diagnosis of IC.

**Figure 1:**
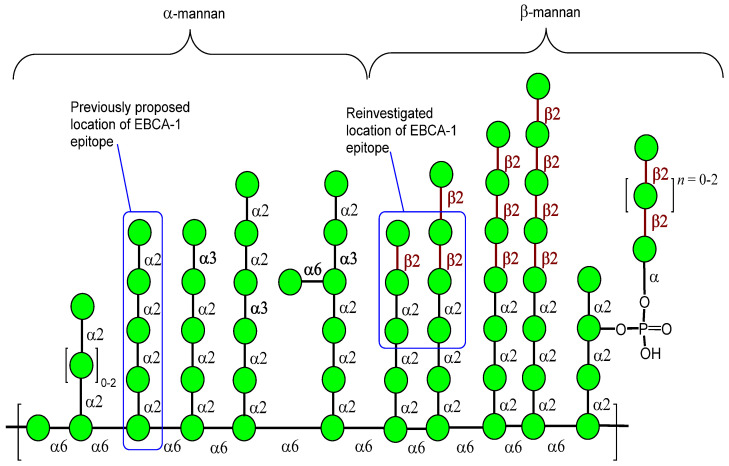
Key structural elements of the mannan of *C. albicans* previously proposed [14,15,16] and reinvestigated location of EBCA-1 epitope.

The native *Candida* mannan is a complex heterogeneous polysaccharide, comprising an α-(1→6)-mannoside backbone and α/β-(1→2)-mannoside side chains of variable lengths and structure (Figure 1). Thus, this polysaccharide may contain a number of other structural elements, including α-(1→3)-linkages, 3,6-branches, and even non-mannose carbohydrate residues. The particular structures of the mannan differ between *Candida* species and depend on a complex mannan biosynthesis network and cell wall remodeling genes [17,18]. The key point for understanding the basis of false-negative results is the elucidation of a precise chemical structure of the EBCA-1 which recognizes epitope within the mannan structure.

In a 1998 study that used oligomannosides prepared by depolymerization of the natural mannan, the minimal epitope of an anti-*Candida albicans* mannan monoclonal antibody EBCA-1 was claimed to be homo-α-(1→2)-mannopentaose [19,20,21]. Herein, we report the reinvestigation of the EBCA-1 carbohydrate specificity using the library of individual synthetic mannooligosaccharides related to the distinct fragments of the *Candida* mannan [22]. Modern methods of carbohydrate chemistry permit efficient and stereospecific assembling of complex oligosaccharide chains representing different parts of polysaccharides of the fungal cell wall [22,23,24]. The arrays of oligosaccharides (glycoarrays) were shown to be indispensable tools in the assessment of ligand specificity of anti-carbohydrate monoclonal antibodies [25,26,27,28,29,30,31] and of antibodies in the blood sera [26,32,33,34].

## 2. Materials and Methods

### 2.1. Biotinylated Synthetic Oligosaccharides

**Figure 2:**
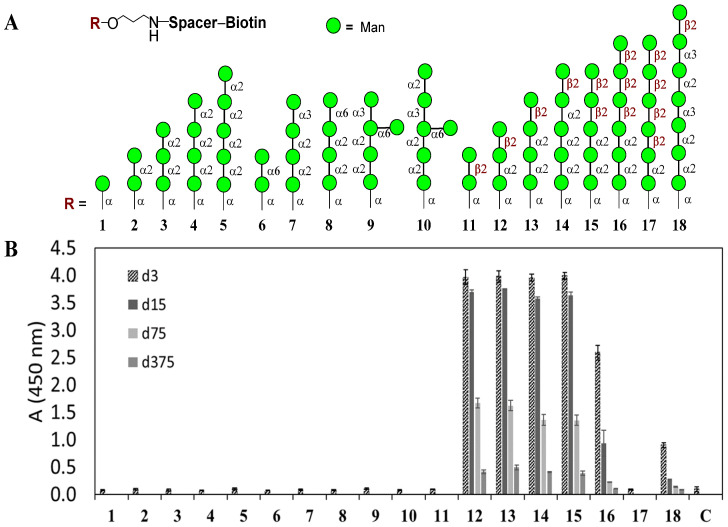
Investigation of oligosaccharide specificity of mAb EBCA-1. (**A**) Composition of thematic glycoarray built up of oligosaccharide ligands representing key structural elements of *C. albicans* mannan, and (**B**) the results of assaying carbohydrate specificity of EBCA-1 with the use of 3-, 15-, 75- and 375-fold dilutions of EBCA-1. C—control, absence of biotinylated ligand in the well.

Biotinylated glycoconjugate ligands 1–18 (Figure 2) [22] were chemically synthesized [23,35,36,37,38,39,40,41] by means of coupling parent aminospacered oligosaccharides with an activated biotin derivative containing a hydrophilic hexaethylene glycol linker [42].

### 2.2. Glycoarray

The wells of 96 streptavidin-coated plates (Thermo Scientific, Rockford, IL, USA.) were coated with biotin-tagged oligosaccharides 1–18 (Figure 2) (100 µL of a 15 pmol/well solution in PBS containing 0.05% Tween-20 and 0.1% BSA) and then incubated for 2 h at 37 °C. After washing, the plates were incubated with a conjugate of EBCA-1 with peroxidase (working solution from the Platelia Candida Ag Kit) in 3-, 15-, 75- and 375-fold dilution for 1 h at 37 °C. After washing five times, color was developed using a TMB monocomponent substrate (100 µL) for 15 min, and the reaction was stopped with 50 µL of 1 M sulfuric acid. Absorbance was measured at 450 nm using a MultiSkan GO plate reader (Thermo Fisher Scientific, Vantaa, Finland). All measurements were performed in triplicate.

## 3. Results

The selection of synthetic oligosaccharide derivatives 1–18 (Figure 2A) for this study was based on the previous structural investigations of *Candida* mannan [17]. Oligosaccharides 1–5 represent fragments of the homo-α-(1→2)-linked mannan chain which corresponds to a so-called antigenic factor 1 [43]. This structural motif is the most typical for mannans from various *Candida* species and strains, and was previously reported as recognized by monoclonal antibody EBCA-1 [19]. Oligosaccharides 6–10 are related to different antigenic factors (4, 13b, 34) present in the yeast cell wall mannan. Oligosaccharides 11–18 belong to the group of β-mannan fragments (antigenic factors 5 and 6), which are very important for host–yeast interaction and the pathogenicity of *Candida*.

The biotinylated conjugates 1–18 were immobilized on the surface of the streptavidin-coated plates to construct the thematic glycoarray. Surprisingly, its use in the reassessment of the carbohydrate specificity of the mAb EBCA-1 (Figure 2) did not detect visible binding with homo-α-(1→2)-linked pentamannoside 5, which was initially proposed by Poulain et al. as the minimal epitope of mAb EBCA-1 [19,20,21]. Very recently, the same authors have reported [44] that mAb EBCA-1 has dual specificity and is able to recognize both homo-α-(1→2)-linked pentamannoside and Man-β-(1→2)-terminated α-(1→2)-linked oligomannoside chains, similar to the ones in glycoconjugates 12–15. Instead, our study only recognized oligomannosides containing a β-glycoside bond, namely compounds 12–15 (and 16 to a lesser extent). Oligosaccharides 12–15 all contain a β-Man-(1→2)-α-Man-(1→2)-α-Man trisaccharide fragment which, thus, can be assigned as a correct minimal holder of the epitope of mAb EBCA-1.

It was noticeable that the elongation of β-Man-(1→2)-α-Man-(1→2)-α-Man trisaccharide sequence from the “reducing” end by one 2)-α-Man unit (12→13) or its dimer 2)-α-Man-(1→2)-α-Man (12→14), or that elongation from the “non-reducing” end by one β-Man-(1→2)-unit (12→15) have not influenced the recognition by mAb EBCA-1. On the contrary, the elongation of the β-Man-(1→2)-α-Man-(1→2)-α-Man trisaccharide sequence from the “non-reducing” end by dimer β-Man-(1→2)-β-Man-(1→2)-(12→16) remarkably decreased the binding with mAb EBCA-1, while the attachment of one additional β-Man-(1→2)- unit (16→17) practically blocked the binding to mAb.

## 4. Discussion

The knowledge of fine carbohydrate specificity of the monoclonal antibody used in a diagnostic kit is important for understanding the molecular basis of observed possible false-positive and false-negative results. Previously, we reported the reinvestigation of carbohydrate specificity of EB-A2 monoclonal antibody used for the immune detection of the *Aspergillus fumigatus* galactomannan [25], where the connection between cross-reactivity, false-positive results, and the specificity of mAb EB-A2 was hypothesized. Herein, we found that the epitope-containing oligosaccharide recognized by the anti-mannan antibody EBCA-1 is the β-Man-(1→2)-α-Man-(1→2)-α-Man trisaccharide only, and not the previously proposed homo-α-(1→2)-linked pentamannoside [19,20,21] or both homo-α-(1→2)-linked pentamannoside and Man-β-(1→2)-terminated α-(1→2)-linked oligomannoside chains. We can only assume that the unusual duality of carbohydrate specificity reported [44] for mAb EBCA-1 could be detected because of the presence of β-Man-contamination in the used homo-α-(1→2)-linked pentamannoside that can be concluded from its published NMR spectra [45].

The reinvestigated trisaccharide epitope is abundantly present in many yeast mannans, however there are *Candida* strains lacking such fragment in the structure of their mannans [46]. This makes it impossible to detect such pathogens using this monoclonal antibody, leading to an increase in false-negative results.

Taking into account the assessed specificity of the EBCA-1 reported here, the binding of this mAb to homo-α-(1→2)-manno-fragments of *Candida* mannans reported previously (see for example papers [19,20,21,47] looks questionable. It should also be noted that the oligosaccharide fragment β-Man-(1→2)-α-Man-(1→2)-α-Man is not dominant in mannan of certain *Candida* strains [46]. It can be capped with two and three β-(1→2)-linked mannoside residues. Such capped sequences (for example in ligands 16 and 17 on Figure 2) have a rather low ability to bind to mAb EBCA-1 due to conformational changes and spatial loop formation reported by Bundle et al. [48].

To the best of our knowledge, there is no diagnostic antibody with an undoubtedly proven ability to recognize homo-α-(1→2)-linked oligomanoside chains. The raising of such mAbs is a rather complex task due to the tolerance of the mammalian immune system to α-mannosides, as opposed to higher immunogenicity of β-mannosides. However, antibodies against α-(1→2)-linked oligomanosides containing branch points of *Candida* mannan would be very promising for high performance diagnostics of invasive candidiasis. The generation of mAbs which are capable of recognizing such fragments are still faced with a challenge to test their applicability for clinical diagnostic needs.
